# Supplementary material for: What Factors Are Associated with Attitudes towards HPV Vaccination among Kazakhstani Women? Exploratory Analysis of Cross-Sectional Survey Data
Source: Vaccines (Basel). 2022 May 23;10(5):824. doi: 10.3390/vaccines10050824 (PMC9146459; doi:10.3390/vaccines10050824)
Supplement: Supplementary file 1 [file vaccines-10-00824-s001.zip › vaccines-1675697-supplementary.pdf]

HPV vaccine questionnaire
Part 1

1. How old are you?________________

2. What is the highest degree of education you've received?
☐ Elementary (1-4 classes)
☐ Middle school (5-9 classes)
☐ High school (10-11 classes)
☐ Secondary technical or vocational
☐ University degree (bachelors)
☐ Master, Ph.D.

3. Your nationality

☐ Kazakh
☐ Russian
☐ Other

4. Where do you live?
☐ Nur-Sultan
☐ Almaty
☐ Aktobe
☐ Oskemen
☐ Pavlodar
☐ Other __________________

5. Your relationship status?
☐ Single
☐ Married
☐ Cohabitating with a partner/constant partner
☐ Divorced
☐ Widowed
☐ Other ______________

6. How many children do you have_________________
7. How many female children do you have_________________
How many male children do you have_________________

8. How old are your children
____

9. What is your monthly family income (tenge)?
☐ ≤ 100 000
☐ 101 000 - 200 000
☐ 201 000 – 300 000
☐ 301 000 – 400 000
☐ ≥ 401 000

CONTEXTUAL FACTORS

1. Have you ever heard about the HPV vaccine?
   a. Yes
   b. No
2. If you answered yes, which sources of information did you rely on to get information about the HPV vaccine?
   a. from the attending physician
   b. from a pediatrician
   c. from a gynecologist
   d. Television
   e. Social networks
   f. Written press
   g. Family
   h. Friends
   i. School
   j. Other
3.

	Please rate to which extent you agree with following statements	Completely disagree	Somewhat disagree	Neutral	Somewhat agree	Completely agree
1.	Because of the controversy surrounding the COVID-19 vaccine, I have less confidence in HPV vaccination recommendations.	1	2	3	4	5
2.	Since the HPV vaccination program launched in Kazakhstan in 2013 has led to complications in some cases, I have less confidence in the healthcare system.	1	2	3	4	5

3.	I think vaccinating teenage girls against HPV encourages them to have sex	1	2	3	4	5
4.	It's hard to talk to my daughter about her sexual health<br>Skip the question if you don't have a daughter/daughters	1	2	3	4	5
5.	I am uncomfortable discussing my daughter's sexual health with a doctor or other healthcare professional<br>Skip the question if you don't have a daughter/daughters	1	2	3	4	5
6.	I am having difficulty bringing up the subject of the HPV vaccine with my daughter<br>Skip the question if you don't have a daughter/daughters	1	2	3	4	5
7.	I am in favor of the compulsory vaccines for children approved by the government of Kazakhstan	1	2	3	4	5
8.	Everyone should be able to decide which vaccines are needed for their children	1	2	3	4	5

4. I happened to refuse a vaccine for my daughter (or choose not to give her a vaccine) Skip the question if you don't have a daughter/daughters
   a. Yes
   b. No
5. I happened to refuse a vaccine for myself
   a. Yes
   b. No
6. I know someone who got seriously ill after getting vaccinated
   a. Yes
   b. No
7. I know a person who became seriously ill because they were not vaccinated
   a. Yes
   b. No
8. Personally, I have already had abnormal cervico-vaginal smears for which treatment was necessary (conization / surgery)
   a. Yes
   b. No
9. Alternative medicines strengthen the body's defenses, thus leading to a complete cure (please rate to which extent you agree with the statement from 1-least to 5-most)

1	2	3	4	5

10. I prefer my daughter to naturally develop defenses against papillomavirus infections rather than through vaccination (please rate to which extent you agree with the statement from 1-least to 5-most). Skip the question if you don't have a daughter/daughters

1	2	3	4	5

11. Have you ever searched for information about the HPV vaccine in the past?
    a. Yes
    b. No
12. (If has already looked for information) Name the 3 most consulted sources of information
    a. Attending physician
    b. Other health professional
    c. Internet
    d. Family
    e. Books
    f. Written press
    g. Other
13. (If used the internet) How did you use the internet?
    a. I consulted forums

    b. I consulted blogs
    c. I consulted social networks
    d. I consulted information sites
    e. Other
14. (If has consulted information sites) Which site?______________
15. (If has already researched information) After having had all this information, were you able to make a decision regarding the HPV vaccination?
    a. Yes, easily
    b. Yes, with difficulty
    c. No, not really
    d. Not at all
16. Papillomavirus infections are very rare
    a. True
    b. False
    c. Don't know
17. Men cannot get papillomavirus infection
    a. True
    b. False
    c. Don't know
18. A person can be infected with HPV for many years without knowing it
    a. True
    b. False
    c. Don't know
19. HPV can be transmitted through oral sex
    a. True
    b. False
    c. Don't know
20. HPV vaccine protects against all sexually transmitted infections
    a. True
    b. False
    c. Don't know
21. HPV vaccine protects against genital warts
    a. True
    b. False
    c. Don't know
22. HPV vaccines are most effective when given to people who have never had sex
    a. True
    b. False
    c. Don't know
23. A person who has been vaccinated against HPV can still develop cervical cancer
    a. True
    b. False
    c. Don't know
24. Girls who have been vaccinated against papillomaviruses need Papillomavirus Pap smears when they are older
    a. True
    b. False
    c. Don't know
25. HPV vaccine helps cure HPV infection
    a. True
    b. False
    c. Don't know
26. Having sex at a young age increases your chances of getting HPV infection
    a. True
    b. False
    c. Don't know
27. Papillomavirus infections can cause throat cancer
    a. True
    b. False
    c. Don't know
28. Please rate the extent to which you trust the following sources to tell the truth about vaccines

	Completely distrust	Somewhat distrust	Neutral	Somewhat trust	Completely trust
Pharmaceutical industry	1	2	3	4	5
government	1	2	3	4	5
your attending physician	1	2	3	4	5
physicians in general	1	2	3	4	5
pharmacists	1	2	3	4	5
other health professionals	1	2	3	4	5
scientific researchers	1	2	3	4	5
traditional media	1	2	3	4	5
alternative media	1	2	3	4	5

29.

	Please rate to which extent you agree with following statements	Completely disagree	Somewhat disagree	Neutral	Somewhat agree	Completely agree
1.	HPV vaccine may be responsible for long-term health problems	1	2	3	4	5
2.	There hasn't been enough research done on the HPV vaccine	1	2	3	4	5
3.	HPV vaccines are not safe	1	2	3	4	5
4.	HPV vaccine is effective in preventing HPV infections	1	2	3	4	5
5.	HPV vaccine is effective in preventing genital warts	1	2	3	4	5
6.	Getting my daughter vaccinated against HPV will help protect her against sexually transmitted infections<br>Skip the question if you don't have a daughter/daughters	1	2	3	4	5
7.	HPV vaccine is effective in preventing cancers associated with HPV	1	2	3	4	5
8.	Using a condom prevents transmission of papillomavirus infections	1	2	3	4	5
9.	Pap smear helps prevent cervical cancer	1	2	3	4	5
10.	Most of my friends get their daughters vaccinated against HPV	1	2	3	4	5
11.	Doctors / healthcare professionals think it's a good idea to get my daughter vaccinated against HPV<br>Skip the question if you don't have a daughter/daughters	1	2	3	4	5
12.	HPV vaccine too new to know if it's safe and reliable	1	2	3	4	5
13.	It would be easier to vaccinate my daughter if the doctor had vaccines in his office to vaccinate my daughter the same day<br>Skip the question if you don't have a daughter/daughters	1	2	3	4	5
14.	If the HPV vaccine was important, it would have been made mandatory	1	2	3	4	5
15.	I think my daughter is too young to be vaccinated against HPV<br>Skip the question if you don't have a daughter/daughters	1	2	3	4	5
16.	HPV vaccine was not made mandatory because it is risky	1	2	3	4	5
